# Integrating Inflammatory, Hemodynamic, and Metabolic Cues: Context-Dependent and Mechanosensitive Regulation of Endothelial Notch Signaling in Cardiovascular Disease

**DOI:** 10.3390/cells15070601

**Published:** 2026-03-28

**Authors:** Genesis Aaliyah Campbell, Omaida Caridad Velazquez, Zhao-Jun Liu

**Affiliations:** 1 DeWitt Daughtry Family Department of Surgery, University of Miami Miller School of Medicine, Miami, FL 33136, USA; gac274@miami.edu; 2 Department of Biochemistry & Molecular Biology, University of Miami Miller School of Medicine, Miami, FL 33136, USA; 3 Department of Radiology, University of Miami Miller School of Medicine, Miami, FL 33136, USA

**Keywords:** endothelial Notch signaling, cardiovascular disease, mechanosensitive signaling, metabolic stress, shear stress, endothelial dysfunction, inflammation, ligand bias, vascular remodeling, context-dependent signaling

## Abstract

**Highlights:**

**What are the main findings?**

- Endothelial Notch signaling regulates vascular identity and angiogenic patterning through mechanosensitive integration of inflammatory, metabolic, and hemodynamic signals.
- In cardiovascular disease, dysregulated endothelial Notch signaling activity arises not solely from uniform activation or suppression but also from disruption of spatial, temporal, and flow-dependent regulatory control, driving vascular remodeling and progression of disease.

**What are the implications of the main findings?**

- Effective therapeutic strategies targeting endothelial Notch signaling should prioritize context-dependent modulation, including ligand-specific regulation and spatial targeting of disturbed hemodynamic flow regions, rather than global pathway inhibition.
- Establishing quantitative and mechanistic parameters defining Notch signal intensity, shear stress responsiveness, and ligand bias will be critical for translating the regulatory role of endothelial Notch into clinically actionable therapies for cardiovascular disease.

**Abstract:**

Endothelial dysfunction is a central feature of cardiovascular disease (CVD) and reflects the maladaptive integration of hemodynamic, metabolic, and inflammatory cues within the vascular microenvironment. The endothelial Notch signaling pathway has emerged as a conserved regulatory pathway whose output is highly dependent on cellular context. While its developmental roles are well established, how endothelial Notch integrates mechanical, inflammatory, and metabolic cues in adult CVD remains incompletely defined. Addressing this gap is essential for understanding why both excessive and insufficient Notch activation are associated with cardiovascular pathology. This review synthesizes current evidence defining the mechanistic regulation of endothelial Notch signaling and contextualizes its dysregulation across major CVD phenotypes, highlighting the need for precision-based therapeutic strategies that restore physiological Notch signaling without global pathway suppression.

## 1. Introduction

Cardiovascular disease (CVD) persists as the leading global cause of mortality, with continued clinical and socioeconomic burden despite ongoing advances in preventative, interventional, and pharmacological target therapies [1,2]. Across diverse clinical manifestations of CVD, including coronary artery disease, cardiomyopathy, atherosclerosis, pulmonary vascular disease, heart failure, and arteriovenous malformations, a unifying feature is endothelial dysfunction [3]. This dysfunction is characterized by inflammatory activation, impaired barrier integrity, maladaptive vascular wall remodeling, and altered vasoreactivity. Rather than functioning merely as a passive lining barrier, in this context, the vascular endothelium operates as an active sensor of biochemical and mechanical stimuli input to regulate gene expression and transcriptional responses that modulate vascular homeostasis. Within the various signaling pathways influencing vascular biology, the Notch pathway is a highly conserved pathway critical for cell fate decisions and developmental patterning in vertebrates [4]. Over the past three decades, numerous studies have established the responsibility of Notch signaling in vascular development and homeostasis, primarily through its regulatory effects on endothelial and cardiac cells, as well as its role in mediating intercellular communication. More specifically, aberrant endothelial Notch signaling has been noted as a direct contributor to CVD pathology [5]. To provide a contextual foundation for how endothelial Notch is regulated in CVD, it is critical to first briefly outline the canonical Notch signaling mechanism. Canonical Notch signaling operates through a series of ligand–receptor interactions between adjacent cells, leading to regulated intracellular signaling that modulates endothelial gene expression and cellular fate.

A major challenge in the field is that endothelial Notch signaling does not function as a simple on/off switch, as suggested by simplified mechanistic models of Notch signaling. Instead, endothelial Notch activity is highly context-dependent and shaped by metabolic stressors, inflammatory cues, and hemodynamic forces [6]. Among these contextual inputs, hemodynamic forces such as blood flow-derived shear stress significantly shape the endothelial phenotype. Laminar shear stress in straight arterial regions maintains an anti-inflammatory atheroprotective transcriptional profile, whereas disturbed shear stress at arterial branches drives pro-inflammatory signaling and increases susceptibility to atherogenesis [7]. Additionally, oxidative stress (OS), hypoxia, inflammatory signaling, and general metabolic shifts can alter expression of the Notch receptors and ligands, as well as post-translational processing and transcriptional output. Despite these findings, the current literature largely positions endothelial Notch signaling within CVD pathology in developmental and genetic paradigms, with limited integration of Notch responses to adult vascular environmental cues, particularly inflammation, metabolism, and mechanotransduction. The current gap in the existing literature limits a comprehensive understanding of how endothelial Notch contributes to the onset and progression of CVD in adult vascular networks. 

In this review, we aim to synthesize published supporting evidence on how the functions of endothelial Notch signaling serve as a mechanosensitive, context-dependent regulator of vascular homeostasis and how disruption or dysregulation of such homeostatic balance contributes to the pathogenesis of CVD. This review will first explore the canonical and non-canonical components of Notch signaling, with a specific focus on the canonical pathway due to its association with CVD when dysregulated. Following this, we will then discuss the mechanosensitive regulation of endothelial Notch under laminar versus disturbed shear stress, noting resulting flow-dependent ligand switching and mechanotransduction pathways. In parallel, the metabolic and inflammatory stressors that modulate endothelial Notch output will be examined, as will how these stressors contribute to pathological endothelial transitional states. This review will conclude by contextualizing endothelial Notch dysregulation across diverse manifestations of CVD, while also examining current therapeutic efforts and ongoing challenges. It will highlight the need for precision-based strategies that account for the tightly regulated physiological parameters of Notch signaling necessary for vascular homeostasis, in which both hyperactivation and suppression contribute to cardiovascular remodeling.

## 2. The Canonical Notch Signaling Pathway in Endothelial Cells

The Notch signaling pathway is a highly conserved, juxtacrine signaling mechanism that regulates cell fate decisions, development, and renewal across multiple tissues. Notch receptors and their ligands are single-pass transmembrane proteins, and thus, signaling is limited to direct cell-to-cell communication, allowing for context-dependent regulation and spatial precision in the vascular wall [8]. During biosynthesis, Notch receptors undergo initial S1 proteolytic cleavage by a furin-like protease, resulting in a heterodimeric receptor that is transported to the cell surface and prepared for ligand-induced activation, enabling rapid and spatially restricted activation in adult endothelial cells [9]. 

Within the vascular system, canonical Notch signaling is central to angiogenic patterning, endothelial differentiation, and vascular homeostasis. Dysregulation of Notch signaling has been implicated in various adult CVD pathologies. At the molecular level, Notch receptor activation is irreversible upon initiation, as it involves proteolytic cleavage, NICD release, nuclear translocation, and DNA-bound protein association [9]. These properties position Notch signaling as a central integrative pathway of local environmental cues, where activation dynamics influence downstream transcriptional responses.

### 2.1. Molecular Mechanism of the Canonical Notch Signaling Pathway

While the influence of endothelial Notch has been comprehensively reviewed in developmental contexts, recent studies have expanded this perspective by demonstrating its sustained role in the maintenance of adult vascular homeostasis, with endothelial Notch expression robustly regulated by physiological and pathological conditions relevant to CVD [4,10,11]. The canonical Notch signaling pathway is initiated when a membrane-bound ligand of the Delta-like (DLL1 and DLL4) or Jagged (JAG1 and JAG2) family on the signal sending cell binds to a Notch receptor on the neighboring cell that is receiving the signal [12]. Ligand-binding from the sending cell to the receptor of the receiving cell induces a conformational change that positions the extracellular juxtamembrane region at the sequential proteolytic cleavage. This process begins with ADAM10, a metalloprotease, mediating proteolytic cleavage at the S2 site, followed by subsequent S3 cleavage by the γ-secretase complex—composed of presenilin, nicastrin, anterior pharynx-defect 1 (APH1a), and presenilin enhancer 2 (PEN2)—resulting in the release of the Notch intracellular domain (NICD) from the membrane [13]. 

Once cleaved, the NICD translocates to the nucleus to bind to the DNA-associated transcription factor, Recombination Signal Binding Protein for Immunoglobulin Kappa J region (RBPJ), displacing repressors and recruiting coactivators such as histone acetyltransferases and Mastermind-like (MAML) proteins [11]. This transition converts RBPJ from a transcriptional repressor to an activator.

When NICD is not present in the nucleus, RBPJ recruits corepressor complexes, including histone deacetylases, to suppress the expression of target genes. The NICD-dependent transcriptional switch induces the expression of canonical Notch target genes, including members of the Hairy and Enhancer of Split (HES) and Hairy/Enhance-of-Split Related with YRPW motif (HEY) families, which function to coordinate angiogenic behavior, junctional stability, inflammatory regulation, and proliferation of endothelial cells.

### 2.2. Endothelial Notch Receptor–Ligand Interactions, Specificity, and Ligand Bias

Despite the various vascular expressions of Notch receptors and ligands, endothelial Notch signaling is largely governed by Notch1 as well as its primary ligands, DLL4 and JAG1. In the context of DLL4-mediated signaling, DLL4 is strongly associated with the stabilization of arterial identity through regulated specification of angiogenic tip-stalk cells and restricting excessive sprouting through the suppression of VEGF responsiveness in adjacent endothelial cells [8,14,15]. Pharmacological inhibition or reduction in DLL4-mediated Notch signaling results in excessive, disorganized angiogenic sprouting, ultimately highlighting the critical role in maintaining vascular integrity [13,14]. In contrast, JAG1 expresses context-dependent activity, acting as a proangiogenic ligand modulator of Notch signaling. Under specific conditions, JAG1 promotes angiogenic expansion, endothelial activation, and plasticity by antagonizing DLL4-mediated lateral inhibition [16]. Recent genetic studies position JAG1 as central to both tip cell formation and endothelial proliferation, with endothelial-specific loss of JAG1 correlating with reduced endothelial cell density and vascular branching during postnatal and embryonic angiogenesis [16].

**Figure 1:**
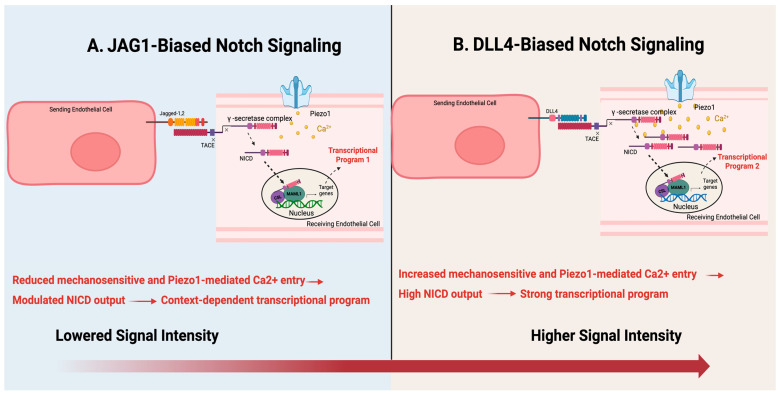
Ligand identity determines mechanosensitive notch signaling amplitude and transcriptional output in endothelial cells. Canonical Notch signaling in endothelial cells is not a binary system but instead acts in both a ligand-dependent and context-sensitive manner. JAG1-biased Notch signaling (**A**) is associated with decreased mechanosensitive Piezo1-mediated Ca^2+^ generation, promoting moderate cleavage of the γ-secretase complex and lower-amplitude NICD production, leading to downstream context-dependent transcriptional programming. The JAG1-biased signaling profile observed supports environmentally responsive and controlled endothelial gene expression. Comparatively, DLL4-biased Notch signaling (**B**) is associated with increased mechanosensitive Piezo1-mediated Ca^2+^ output and further supports robust activation of the γ-secretase complex, high-amplitude NICD generation, and dominant transcriptional activation, in which stronger signaling states drive further amplified downstream gene expression programs. The lowercase ‘x’ denotes the site of TACE-mediated proteolytic cleavage in the Notch signaling pathway. Figure created with Biorender.com.

Notch output is further refined by fringe family glycosyltransferases, which modify both the extracellular domain of the Notch receptor and responsiveness of biased ligands. At the mechanistic level, JAG1 functions as a competitive Notch ligand, counteracting DLL4-mediated receptor binding; however, its signaling capacity is diminished in endothelial cells expressing Fringe family glycosyltransferases, which preferentially enhance DLL4-Notch interactions while attenuating JAG1-mediated signaling [16]. This differential modulation establishes a ligand-dependent signaling hierarchy that modulates overall signaling intensity and downstream transcriptional programs in response to surrounding vascular environment (Figure 1) [8,16]. The ligand-specific regulation reinforces the notion that endothelial Notch signaling functions on a graded spectrum as compared to a binary on/off switch in adult cardiovascular tissue, thereby promoting angiogenic plasticity and endothelial specification. The homeostatic balance between DLL4 and JAG1 signaling must be carefully regulated to maintain physiological angiogenesis; while loss of JAG1 prevents uncontrolled sprouting, spatially unbalanced or excess expression of JAG1 can alter the tip-stalk pattern and further drive pathological angiogenesis [17]. 

Such findings underscore that endothelial Notch signaling is governed by distinct ligand-specific properties—including spatial distribution, relative abundance, and biochemical modulation of ligand–receptor interactions—rather than by receptor activation alone within the vascular microenvironment. Emerging evidence further refines this framework, suggesting that DLL4 and JAG1 differ not only in signaling strength but also in temporal activation dynamics. Using bioengineered ligand micropatterns, DLL4-functionalized substrates more efficiently directed endothelial sprout spatial orientation and positioning, whereas comparatively JAG1-mediated substrates required prolonged exposure to achieve comparable effects of spatial behavior control [18,19]. Computational modeling further demonstrated that distinct DLL4- and JAG1-mediated sprouting responses correlate with ligand-specific Notch activation kinetics, where differences in the rate and duration of receptor activation regulate endothelial sprouting behavior, spatial orientation, and signaling outcomes.

Endothelial Notch signaling does not produce single, uniform outcomes but instead operates as a ligand-biased system where signaling intensity, timing, and transcriptional output are largely influenced by receptor modification, signaling kinetics, and spatial organization within the vascular environment. The differences noted in the temporal dynamics, particularly those observed in DLL4 and JAG1, drive differential endothelial responses during angiogenesis, highlighting the overall signaling kinetic and environmental context in determining Notch transcriptional outcomes. The observed ligand-dependent fine-tuning demonstrates a mechanistic framework where Notch integrates with adjacent endothelial signaling pathways to collectively modulate vascular behavior.

### 2.3. Integrative Crosstalk and Coordination Between Notch Signaling and Core Endothelial Pathways

Signaling initiated through the canonical Notch pathway relies on continuous and dynamic integration with parallel endothelial signaling pathways and vascular microenvironmental cues, positioning Notch as a context-dependent regulatory modulator rather than an autonomous linear cascade [10]. In endothelial cells, activity of the canonical Notch signaling pathway is influenced by densely interconnected crosstalk with pathways that regulate metabolism, mechanotransduction, inflammation, angiogenesis, and cellular plasticity [10]. Integration of parallel endothelial signaling pathways allows for coordinated transcriptional outcomes responsible for vascular maintenance and stabilization, while enabling adaptive responses to physiological stress. As the canonical Notch pathway incorporates crosstalk across extensive endothelial pathways, endothelial Notch signaling regulates and fine-tunes responses to target genes as well as biochemical and mechanical stimuli, preserving vascular homeostasis and minimizing maladaptive remodeling.

Among these interactions, the interplay between Notch and vascular endothelial growth factor (VEGF) is the most comprehensively understood example of this regulatory role. DLL4-Notch1 signaling functions as a negative feedback mechanism that constrains VEGF-driven angiogenesis through the suppression of VEGFR2 expression in neighboring cells, preventing excessive endothelial sprouting and preserving organized vascular patterns [15,20]. In the context of Notch and VEGF interplay, Notch does not initiate angiogenesis but instead modulates spatial distribution and organization of angiogenic sprouting, thereby ensuring proportional VEGF responsiveness to physiological demand. In addition to VEGF, Notch signaling integrates with the PI3K/Akt pathway to influence both metabolic and survival state of endothelial cells [20].

Furthermore, Notch signaling crosstalks with bone morphogenetic proteins/transforming growth factor beta (BMP/TG-β) pathways to regulate endothelial plasticity and structural integrity, including endothelial-to-mesenchymal cell transition (EndMT) under pathological conditions and stressors [21]. Through these interactions, Notch facilitates the maintenance of endothelial stability while maintaining response to disease-relevant stress signals. In parallel, Notch signaling also interacts with other developmental signaling pathways retained in the adult vascular endothelium, such as Wingless/Integrated signaling pathway (Wnt) and Sonic hedgehog through direct cell–cell communication to coordinate endothelial fate decisions as well as vascular remodeling in both contexts [22]. While the Wnt signaling pathway is responsive to mechanical cues in certain tissues, it does not function as a primary endothelial mechanosensor and instead is largely associated with downstream mechanotransductive inputs, where its interaction with Notch modulates proliferative capacity, endothelial stability, differentiation, and phenotypic response under physiological and pathological conditions. Dysregulation of Wnt-Notch coupling has been shown to link to endothelial dysfunction and maladaptive vascular remodeling across multiple CVD contexts [22].

These interactions further reinforce Notch as a stabilizing buffer that mediates endothelial responses to metabolic or inflammatory perturbations, thus limiting sustained phenotypic shifts that lead to compromised vascular integrity. Notably, Notch signaling additionally interfaces with pathways associated with sensing and transducing mechanical input. Crosstalk with the Hippo pathway transcriptional co-regulator yes-associated protein (YAP) and the transcriptional co-activator with PDZ-binding motif (TAZ) integrates cell density signals with the transcriptional output, while hemodynamic forces are transmitted through various flow-dependent transcription factors, including Krüppel-like factor 2 (KLF2) and Krüppel-like factor 4 (KLF4), to modulate the activity of the Notch pathway [6,23]. These interacting factors govern transcriptional regulatory networks responsible for anticoagulant function, endothelial barrier integrity, and vascular tone. 

Evidence for the necessity of integrated and organized mechanotransductive regulatory control is provided by inducible, endothelial-specific deletion of KLF2 and KLF4 in adult mice, which results in acute vascular failure characterized by heart failure, myocardial infarction, and stroke. The resulting vascular collapse and pathology are consistent with significant disruption of endothelial barrier function and homeostatic balance, underscoring the critical role of mechanosensing in maintaining vascular integrity [24]. In this context, Notch signaling interacts with flow-responsive transcription factors to preserve vascular stability in adult organisms by facilitating the translation of biochemical and mechanical signals into stable homeostatic outputs, as compared to maladaptive remodeling. Dysregulation or disruption of this integrative regulatory capacity is increasingly recognized as a contributing factor in CVD states characterized by metabolic stress, sustained inflammation, and disturbed hemodynamic flow patterns.

## 3. Mechanosensitive Regulation of Endothelial Notch Signaling

**Figure 2:**
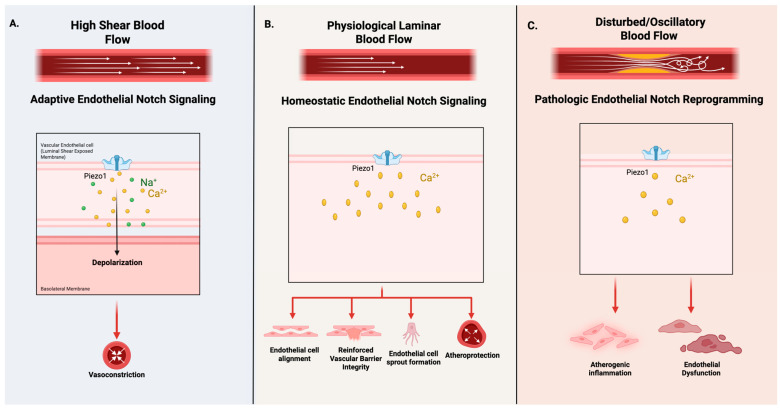
Implication for Notch activation: hemodynamic flow patterns across distinct vascular endothelial shear stress states. Distinct blood flow patterns exhibit functionally distinct endothelial phenotypes and transcriptional outputs as determined by the local microvascular environment. In high-shear adaptive blood flow (**A**), endothelial cells exhibit mechanosensitive activation that maintains membrane polarity and adaptive signaling responses. In physiological states of laminar blood flow (**B**), signaling functions to sustain endothelial stability, barrier integrity, coordinated endothelial sprouting, and protective capacity. Comparatively, oscillatory or disturbed shear blood flow states (**C**) functionally reprogram Notch signaling, resulting in compromised downstream outputs contributing to endothelial cell dysfunction, maladaptive vascular remodeling, and atherogenic inflammation. Luminal and basolateral endothelial membranes are indicated to underscore the preserved cellular polarity across distinct shear states despite varying phenotypic outcomes. Figure created with Biorender.com.

Endothelial cells are continuously exposed to varying mechanical forces at the vessel wall, including circumferential stress from pulsatile vascular pressure, normal stress exerting force perpendicular to the endothelial surface, and wall shear stress resulting from blood flow. Hemodynamic forces are detected through specific endothelial mechanosensors, including vascular endothelial cadherin (VE-cadherin)-based junctional complexes, context-dependent ion channels such as Piezo1, and the glycocalyx, which are transduced into intracellular signaling pathways that facilitate calcium influx and KLF2/4-dependent transcriptional processes [25]. Hemodynamic flow-dependent forces are regarded as critical regulators of endothelial phenotype, shaping transcriptional programs that modulate vascular stability, structural remodeling, and inflammation. In vivo, endothelial cells are subjected to a range of heterogeneous vascular flow environments, ranging from uniform laminar shear stress in straight arterial segments to disturbed flow at vascular curvature regions, bifurcations, and vessel branch points (Figure 2) [6,12]. 

These mechanical inputs, encompassing shear stress magnitude, cyclic stretch, and flow directionality, are actively transduced into intracellular signaling responses that function to regulate endothelial homeostasis and susceptibility to CVD [6,12]. Increasing evidence suggests endothelial Notch signaling as a central component of this mechanotransductive framework, linking mechanical flow-dependent cues to transcriptional programs for stabilization of vascular endothelial function.

### 3.1. Laminar Shear Stress Induces Activation of Notch1 Signaling in Endothelial Cells

Sustained unidirectional laminar shear stress promotes an atheroprotective endothelial phenotype characterized by increased resistance to pathological remodeling, suppression of inflammatory signaling, and reinforced barrier integrity. Under these physiological conditions, endothelial Notch signaling is selectively activated and further aligned with transcriptional programs responsible for vascular stabilization. Current evidence links laminar shear stress to enhanced Notch activation and further downstream expression of canonical target genes, thereby positioning Notch signaling as an established flow-responsive regulator of endothelial homeostasis [6]. 

On a functional level, laminar shear-induced Notch activity is necessary for maintenance of endothelial quiescence, limiting excessive angiogenic activation and cell proliferation. In parallel, Notch signaling promotes an anti-inflammatory endothelial state by suppressing pro-inflammatory gene expression and preservation of intercellular junctional integrity. Loss of endothelial Notch under laminar flow conditions is associated with increased inflammatory transcription and compromised barrier integrity. Together, Notch-dependent responses under laminar flow conditions align with the optimization tight junction organization and reinforced cell–cell adhesion observed under laminar flow, subsequently leading to improved endothelial barrier function [6]. Mechanistically, laminar shear stress aligns Notch signaling with flow-responsive transcription factors, KLF2 and KLF4, which facilitate the endothelial adaptation to shear. Through this coordinated regulatory framework, Notch is enabled to translate mechanical input into transcriptional output that maintains junctional integrity, production of nitric oxide, and anticoagulant signaling, thus supporting vascular endothelial homeostasis. 

### 3.2. Irregular Hemodynamic Flow Drives Endothelial Notch Signaling Toward Pro-Inflammatory Phenotypes

Comparatively, disturbed hemodynamic flow patterns at branch points, vessel curvature, and arterial bifurcations are largely associated with endothelial dysfunction and pathological Notch signaling. Affected regions exhibit dysfunctional Notch signaling dynamics, where normal atheroprotective activity is suppressed or dysregulated, leading to a shift toward pro-inflammatory transcriptional programs. In disturbed flow conditions, the suppression of regulating Notch signaling occurs in conjunction with endothelial activation, marked by impaired barrier function, increased inflammatory signaling, lipid retention, and susceptibility to the recruitment of leukocytes. The current literature has indicated that altered hemodynamic flow disrupts normal physiological outputs of Notch signaling, thereby facilitating phenotypic transitions in the endothelium that increase risk of atherosclerotic lesion development within the vessel wall [26]. Further computational and in vivo analysis support a correlation between Notch dysregulation associated with disturbed hemodynamic flow and the early formation of atherosclerotic plaque [27].

### 3.3. Piezo1-Mediated Mechanotransduction of Shear Stress and Notch1 Signaling

The mechanosensitive ion channel Piezo1 has been identified as a key upstream regulator that couples hemodynamic mechanical shear stress to endothelial Notch activation. By directly sensing these hemodynamic forces, Piezo1 detects mechanical compromise of the endothelial membrane and initiates calcium-dependent signaling cascades that modulate the canonical Notch pathway activity. The recent literature suggests that Piezo1-mediated calcium entry activates the metalloprotease ADAM10, a critical sheddase responsible for S2 cleavage of Notch1, followed by γ-secretase-dependent S3 cleavage, and releases the NICD into the nucleus. In this coupling, endothelial cells translate generated hemodynamic forces into Notch-dependent transcriptional outputs that modulate homeostasis, junctional stability, and inflammatory signaling. In vivo, endothelial-specific disruption of Piezo1 suppresses expression of Notch target genes, ultimately reinforcing the importance of the Piezo1-ADAM10-Notch1 relationship in sustaining Notch signaling under physiological hemodynamic flow states [26,28]. Collectively, these findings position Piezo1 as a critical mechanosensor that confers shear sensitivity to the canonical Notch pathway, thereby positioning Notch1 as a critical influence of endothelial mechanosensitive signaling networks.

### 3.4. Influence of Flow-Dependent Ligand Bias on Angiogenesis

In addition to regulating Notch1 activation through mechanisms of mechanotransduction, hemodynamic forces also influence the qualitative signaling of Notch1 through ligand bias engagement. In laminar flow environments, endothelial cells preferentially engage with DLL4, promoting vascular stabilization, limited angiogenic sprouting, and junctional stabilization. Comparatively, disturbed hemodynamic flow is associated with increased JAG1 dominance, thereby resulting in shifts within ligand expression that alter Notch signaling output, leading to less effective barrier protective transcriptional programs and endothelial stabilization [16]. 

It is important to note that DLL4- and JAG1-mediated Notch signaling are not functionally equivalent and produce distinct outcomes within the endothelium. DLL4-mediated Notch signaling results in strong lateral inhibition and robust suppression of endothelial tip cell behavior, whereas mediation via JAG1 signaling reduces lateral inhibition, permitting endothelial activation and angiogenic sprouting. Flow-dependent ligand bias therefore results in spatially confined variations in Notch signaling strength, duration, and downstream transcriptional output, thus promoting regional heterogeneity in endothelial behavior across arteries, branchpoints, and microvascular bends [16]. Through ligand bias, endothelial cells translate mechanical cues at both activation and signaling output levels, enabling tuning of Notch signaling outputs toward favored vascular remodeling or stabilization.

### 3.5. Proposed Conceptual Model: Endothelial Notch Signaling as a Mechanosensitive Regulator Within CVD

**Figure 3:**
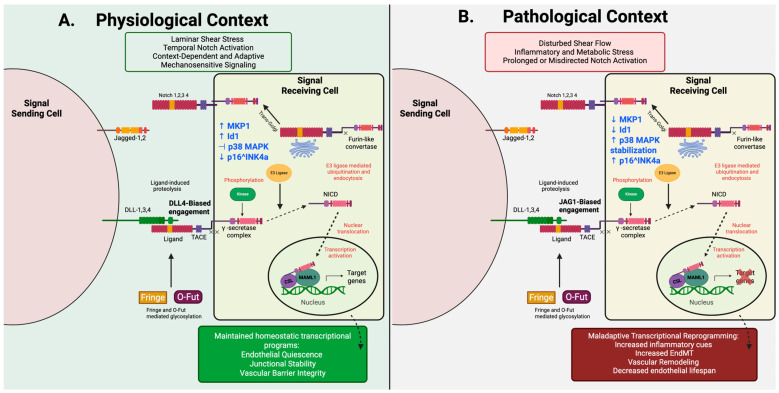
CVD context-dependent regulation of endothelial Notch signaling in physiological and pathological setting. This schematic illustrates the dynamic integration of biomechanical and metabolic cues to yield distinct vascular outcomes. In physiological conditions characterized by laminar blood flow (**A**), endothelial Notch signaling is transiently activated and regulated through mechanosensitive cues with preferential DLL4-biased ligand engagement. This biased engagement promotes induction of MKP1 and Id1, setting forth a signaling cascade that suppresses p38 MAPK activity and disrupts p16^INK4a^ regulation, as well as promote homeostatic transcriptional output that functions to maintain vascular barrier integrity and stability and enhance endothelial quiescence. Comparatively, pathological conditions (**B**) are associated with disturbed shear, metabolic, and inflammatory stress that preferentially favor mislocalized or sustained Notch activation through JAG1-biased ligand engagement. This shift prompts the reduction in MKP1 and Id1 expression, stabilization of p38 MAPK, p16^INK4a^ induction, and reprogramming of transcriptional outputs to favor increased EndMT, vascular remodeling, and inflammation. This schematic is partially adapted from [10], with appropriate modifications to reflect endothelial Notch-specific signaling. The lowercase ‘x’ denotes the site of TACE-mediated proteolytic cleavage in the Notch signaling pathway. Figure created with Biorender.com.

Available synthesized evidence supports a model in which endothelial Notch signaling functions as a dynamic, context-dependent, mechanosensitive regulatory network rather than a binary on/off pathway. Notch1 signaling activity is continuously modulated by both flow patterning and hemodynamic force magnitude, further refined by metabolic and inflammatory cues, enabling proportional regulation of transcriptional programs, including those that maintain inflammatory activation, barrier integrity, and homeostatic balance in the endothelium. Disruption of this regulatory balance, through failure of contextual calibration, rather than complete pathway loss, uncouples Notch signaling from its role of mechanosensitive protection and shifts endothelial states toward maladaptive phenotypes associated with CVD (Figure 3).

## 4. Endothelial Notch Signaling in Metabolic and Inflammatory Regulation

While endothelial Notch signaling promotes vascular homeostasis in physiological conditions, chronic metabolic stress and inflammation significantly alter regulatory function. In the pathological context, Notch signaling is increasingly influenced by cytokine exposure, metabolic reprogramming, hypoxic signaling, and OS, redirecting transcriptional output from endothelial barrier stabilization and quiescence toward endothelial activation, OS, and maladaptive remodeling. Under chronic metabolic stress, endothelial Notch signaling is maladaptively engaged with inflammation pathways that promote vascular dysfunction. Converging evidence indicates that inflammatory mediators such as TNF-α and IL-1β directly influence the modulation of Notch receptor activity and downstream transcriptional programs through NF-kB-dependent mechanisms, establishing a mechanistic relationship between endothelial Notch regulation and innate immune signaling [29]. 

In conjunction, metabolic stress alters engagement of the Notch pathway through modulation of transcriptional programs responsible for glycolysis and mitochondrial function, reinforcing Notch as an active regulator of endothelial metabolic reconstruction in pathological conditions [30]. Beyond inflammatory and metabolic inputs, OS and hypoxia are evidenced as significant amplifiers of endothelial Notch signaling through ROS-dependent mechanisms and HIF-1α-mediated transcriptional crosstalk, promoting a sustained pathway activation in disease contexts [31,32]. Collectively, these models of stress-dependent Notch signaling mechanistically link metabolic imbalance and prolonged inflammation to endothelial dysfunction and progressive vascular remodeling, including EndMT, fibrosis, and vascular wall stiffening, major characteristic of CVD and its progression [33]. The following sections will closely examine the influence of inflammatory, metabolic, and hypoxic stressors that activate endothelial Notch signaling to drive pathological vascular phenotypes.

### 4.1. Inflammatory Modulation of Endothelial Notch Signaling

Within inflammatory microenvironments, endothelial Notch signaling is fundamentally reconfigured through cytokine-driven crosstalk with NF-κB, in turn altering transcriptional output, ligand expression, and endothelial phenotype. In normal physiological context, basal Notch activity contributes to endothelial vascular integrity; however, exposure to pro-inflammatory cytokines shift endothelial identity and function to an activated state. Pro-inflammatory cytokines, such as tumor necrosis factor-α (TNF-α) and interleukin-1β (IL-1β), act as modulators of endothelial Notch pathways through bidirectional coupling with nuclear factor-κB (NF-κB), a critical transcriptional regulator of inflammatory gene expression and innate immune signaling [29]. 

Alongside these modulatory mechanisms, cyclooxygenase (COX) enzymes, particularly cyclooxygenase-2 (COX-2), function as critical mediators of inflammatory signaling within the vascular endothelium. COX-derived prostanoids, such as thromboxane and prostacyclin, serve to regulate platelet activity, vascular tone, and endothelial homeostasis through tightly controlled context-dependent enzymatic balance [34]. In pathological and inflammatory conditions, expression of COX-2 is upregulated, resulting in an increased production of vasoconstrictive and pro-inflammatory mediators that directly contribute to endothelial dysfunction and unregulated vasodilation responses [35]. Disruption of the prostacyclin–thromboxane balance has been noted to promote pro-thrombotic and pro-inflammatory endothelial states within vascular pathology [34]. Recent evidence suggests that COX-dependent inflammatory signaling crosstalks with transcriptional and redox-sensitive pathways that converge with Notch-mediated regulatory networks, including NF-κB-dependent mechanisms. In this context, COX-2-derived prostanoids can influence Notch signaling output through modulation of the endothelial activation state, thus reinforcing a feed-forward loop that amplifies inflammatory signaling and maladaptive vascular remodeling in CVD [35].

Beyond cytokine-driven activation, experimental evidence demonstrates that NF-κB activation induces expression of Notch ligands, notably JAG1 and DLL4, with subsequent enhancement of NICD-dependent transcriptional activity in endothelial cells. This reciprocal interaction enables Notch signaling to reinforce NF-κB-dependent gene expression, establishing a feed-forward signaling loop that functions to stabilize inflammatory endothelial states [29,36]. In the vascular endothelium, convergence of these signaling pathways promotes leukocyte adhesion and junctional destabilization, resulting in increased vascular permeability that is characteristic of endothelial dysfunction across heart failure, atherosclerosis, and systemic inflammatory diseases. Under conditions of prolonged inflammatory exposure, endothelial Notch signaling transitions from an adaptive stabilizing role to a pathological amplifier, where cytokine-driven Notch signaling amplifies inflammatory gene expression, erodes the integrity of the endothelial barrier, and promotes a permissive environment for chronic vascular inflammation. The exhibited maladaptive reprogramming from physiological signaling conditions constitutes a mechanistic link between inflammatory stress and the progression of CVD.

### 4.2. Notch-Dependent Endothelial Reprogramming in Metabolic Stress

Metabolic stress represents a critical regulatory axis influencing endothelial identity, function, and phenotypic expression within CVD. A growing body of evidence has identified Notch signaling as a key regulator of endothelial metabolic programming, establishing a link between utilization of cellular energy and both adaptive and maladaptive vascular response. Endothelial cells predominantly rely on glycolysis for generation of ATP to meet energetic demands, with alterations in metabolic state exerting a direct influence on transcriptional programs governing proliferation, barrier integrity function, and cell survival. Given this glycolytic dependency, pathways that regulate glycolytic flux hold significant influence on endothelial function and adaptation to cellular stress. In this context, Notch signaling has been noted as a potent modulator of endothelial metabolism, with a direct influence on glycolytic gene expression and metabolic flux. Initial insight into this regulatory axis was posited in development studies demonstrating metabolic state and Notch signaling coordination with cell fate decisions [30]. 

Recent publications have further extended these findings to adult endothelial cells, revealing that under stress, Notch signaling regulates metabolic adaptation through the attenuation of nutrient transporters, glycolytic enzymes, and mitochondrial function [37]. Under conditions of sustained metabolic stress and overload, as occurs in chronic hyperglycemia, insulin resistance, and dyslipidemia, persistent Notch activation drives a shift toward a pro-inflammatory and stress-responsive state. In states of CVD characterized by chronic metabolic imbalances, what is normally an adaptive regulatory framework becomes chronically engaged and thus positions Notch-mediated metabolic integration as a determinant of endothelial dysregulation. Progressive and sustained Notch signaling under conditions of chronic metabolic imbalance contributes to progressive vascular pathology, impaired vasoreactivity, and increased inflammatory susceptibility, with effects particularly relevant in linking metabolic stress to CVD states.

### 4.3. Oxidative Stress, Hypoxia, and Persistent Activation of Endothelial Notch Signaling

As a downstream consequence of chronic metabolic imbalance, OS and hypoxia emerge as convergent drivers of sustained endothelial Notch activation. Within the vascular endothelium, the generation of pathological reactive oxygen species (ROS), primarily via NADPH oxidase complexes such as Nox2 and Nox4, functions not only as a non-specific byproduct but also as signaling mediator regulating pathways, including Notch and Wnt/β-catenin, promoting sustained activation of pathways under pathological conditions [31]. The effects of ROS on endothelial Notch signaling are context-dependent. While the generation of transient or spatially restricted ROS can promote adaptive Notch activation that supports both stress tolerance and survival of endothelial cells, in prolonged inflammatory and metabolic states, chronic redox imbalance further reinforces endothelial Notch activation and shifts signaling dynamics towards pathological outcome. 

System-level analysis of cardiovascular redox signaling, including network-based signaling models, transcriptomic profiling, and pathway enrichment, identified ROS as a central convergence point linking Wnt/β-catenin pathways and Notch signaling through redox-dependent modulation of shared transcriptional regulators, co-activator complexes, proteolytic enzymes, and pathway intermediates [31]. The Wnt signaling pathway has a broad influence on embryonic development and adult tissue homeostasis and therefore does not function as a primary sensor of OS within the endothelium. Instead, Wnt signaling operates downstream from redox-mediated signaling imbalance, where it stabilizes β-catenin and sustains NICD-dependent transcriptional activity following the initiation of Notch signaling, thus extending the duration of its transcriptional output under sustained endothelial stress states. While the Wnt/β-catenin signaling pathway maintains transcriptional outputs downstream from redox imbalance, hypoxic stress further amplifies this pathological reinforcement through the direct stabilization of core components of the Notch signaling cascade. Mechanistically, in hypoxic conditions, HIF-1α directly associates with the NICD, increasing nuclear stability to protect it from degradation while promoting sustained assembly of the NICD-RBPJ transcriptional complex at Notch target gene promoters. Through this HIF-1α and NICD interaction, Notch-dependent transcriptional activity is prolonged beyond its normal canonical signal termination window [32]. In acute ischemic settings, this hypoxia-driven stabilization of Notch signaling facilitates endothelial survival and angiogenic adaptation; however, in states of persistent hypoxia, characteristic of ischemic myocardium, fibrotic vascular remodeling, and advanced atherosclerosis, sustained activation leads to maladaptive outcomes and contributes to progressive CVD. 

Emerging integrative reviews of CVD mechanisms further underscore that dysregulation of Wnt-Notch coupling in sustained hypoxic and OS promotes maladaptive vascular remodeling, endothelial dysfunction, and pathological progression across myocardial infarction, atherosclerosis, and heart failure [22]. In this framework, hypoxia-induced Notch stabilization is initially adaptive; however, in states of chronic metabolic and ischemic stress, NICD-dependent transcriptional activity persists. Such prolonged signaling states engage downstream transcriptional programs that facilitate endothelial phenotypic plasticity and vascular remodeling including Notch-mediated EndMT.

In addition to ROS-mediated signaling, nitric oxide (NO) serves as a critical redox-sensitive regulator of endothelial function that interfaces with the Notch pathway. Endothelial nitric oxide synthase (eNOS)-derived NO modulates vascular homeostasis by promoting vasodilation, preserving endothelial quiescence, supporting angiogenic signaling and endothelial repair, and suppressing inflammatory activation [38]. Comparatively, inducible nitric oxide synthase (iNOS) maintains upregulation under inflammatory and pathological conditions, contributing to OS, excessive NO production, impaired endothelial cell function, and dysregulated angiogenesis [39]. Evidence further supports that endothelial Notch signaling converges with NO signaling pathways through homeostatic regulation of redox balance and NOS activity, with NO bioavailability influencing the stability of NICD and subsequent transcriptional output. This characteristic positions Notch signaling as a central link to both protective eNOS-mediated vascular responses and pathological iNOS-mediated endothelial dysfunction within CVD [40].

Alongside nitric oxide-mediated regulation, additional redox-active signaling mechanisms contribute to the context-dependent fine-tuning of endothelial Notch activity. Hydrogen sulfide (H_2_S), another imperative redox-active signaling molecule within vascular biology, is regarded as an important regulator of endothelial function and redox homeostasis. H_2_S exerts vasoprotective effects through the promotion of angiogenesis, attenuation of OS, and preservation of endothelial barrier integrity. Within endothelial systems, H_2_S signaling promotes cellular survival and regenerative capacity by homeostatic modulation of pro-angiogenic pathways and mitochondrial function while simultaneously diminishing inflammatory signals and endothelial dysfunction [41]. At the mechanical level, recent evidence highlights the role of H_2_S in communicating with key developmental signaling pathways, including Notch, through post-transcriptional and redox-dependent regulatory mechanisms. H_2_S-mediated signaling has been noted to modulate endothelial signaling networks that intersect with Notch activity, including endothelial proliferation, angiogenic signaling, and EndMT, all of which are pathways indispensable to both vascular remodeling and the progression of CVD and thereby reinforce its role as a key regulator within this framework [42].

### 4.4. Noncoding and MicroRNA-Mediated Regulation in Endothelial Notch Signaling

Endothelial Notch signaling is further regulated at the post-transcriptional level, through noncoding RNAs, particularly microRNAs, which fine-tune endothelial Notch signaling in response to environmental cues. MicroRNAs function as regulators that modulate Notch receptor expression, ligand availability, and NICD-dependent transcription, coordinating metabolic, inflammatory, and redox-derived signals within the vascular microenvironment [43]. Several microRNAs have been identified as key regulators of endothelial Notch signaling, with each exerting distinct influences on vascular homeostasis and disease progression [44,45,46,47,48]. Among these microRNAs, miR-126, a well-characterized endothelial-specific microRNA, serves a critical role in supporting vascular integrity and angiogenic signaling through the regulation of VEGF and PI3K signaling pathways; it has also been shown to promote proliferation, migration, and survival of endothelial cells through the modulation of Notch1 signaling [44]. In contrast, miR-34a contributes to endothelial senescence and cardiovascular dysfunction through Notch1 signaling suppression, resulting in reduced NICD-dependent transcriptional activity required for endothelial survival, proliferative capacity, and repair, thereby linking vascular aging and decline to impaired Notch function and diminished endothelial repair capacity [45,46]. Building on these regulatory roles, miR-21 has emerged as a critical mediator of fibrotic and inflammatory signaling within the cardiovascular system, contributing to vascular remodeling and fibrosis, with evidence also linking miR-21-associated effects to pathway interactions intersecting with Notch signaling [48]. These findings position microRNAs as critical post-transcriptional regulators of endothelial Notch signaling, bridging environmental stressors to sustained transcriptional reprogramming and reinforcing the context-dependent vascular responses in CVD [44,45,46,47,48].

### 4.5. Vascular Remodeling and Notch-Mediated Endothelial–Mesenchymal Cell Transition

As persistent inflammatory, metabolic, and hypoxic stressors enforce sustained activation of endothelial Notch signaling, downstream consequences can also include EndMT, where endothelial cells undergo a form of pathological phenotypic reprogramming in which there is a progressive loss of endothelial identity and acquisition of mesenchymal features that promote vascular remodeling [33]. Notch signaling has emerged as a critical regulator of EndMT, characterized by the downregulation of endothelial markers, including VE-cadherin, CD31/PECAM-1, vWF, CD34, and the tyrosine kinase receptors TIE1 and TIE2, alongside the induction of mesenchymal gene programs and the acquisition of migratory, contractile, and fibrogenic properties [33,49,50]. 

EndMT is not governed by Notch signaling alone but also through collaborative signaling of both Notch and transforming growth factor-β (TGF-β), a canonical driver of the differentiation of mesenchymal cells and fibrogenic reprogramming. Although transient activation of EndMT associative signaling may support adaptive vascular repair, persistent Notch signaling activation under pathological conditions reinforces TGF-β transcriptional programs and drives irreversible fibrotic remodeling, extracellular matrix deposition, and increased vessel stiffening. The functional interplay between Notch and TGF-β signaling further amplifies EndMT responses through the reinforcement of irreversible vascular remodeling processes and mesenchymal differentiation. Collectively, such findings indicate that EndMT is representative of a late-stage consequence of persistent Notch-driven remodeling processes, underscoring a critical shift in endothelial Notch function toward pathological reprogramming, contributing to structural deterioration of the vascular wall, compromised endothelial barrier integrity, and reduced vascular compliance.

### 4.6. Implication in Progression of CVD

In CVD progression, the cumulative effects of metabolic imbalance, inflammation, OS, and endothelial reprogramming establish dysregulated endothelial Notch signaling as a major determinant of cardiovascular pathology. In states of chronic cellular stress conditions, prolonged Notch activation contributes to endothelial dysfunction, fibrotic remodeling, vascular plaque development, and stiffening, all of which are prominent pathological hallmarks underlying hypertensive vascular diseases, atherosclerosis, and heart failure [22,31,33]. Endothelial Notch signaling should not be viewed as an isolated pathogenic pathway but instead as a core integrative regulatory hub that coordinates metabolic, inflammatory, oxidative, and biomechanical cues from the surrounding microenvironment into durable transcriptional programs regulating endothelial cell identity, plasticity, and end fate. 

Disruption of the normal contextually sensitive temporal precision of Notch activity reorients the pathway toward maladaptive signaling states that reinforce pro-fibrotic, pro-inflammatory, and vascular barrier destabilization, which promote sustained pathological remodeling and further drive progression of CVD. In a translational context, the collective insights challenge the position of Notch signaling as a binary therapeutic target and instead favor a mechanosensitive-dependent modulation of endothelial Notch activity in which activity is selectively modulated to reestablish regulatory signaling balance. Thereby, therapeutic strategies should focus on context-dependent modulation of endothelial Notch activity, rather than complete global pathway inhibition, to restore regulatory balance while preserving the adaptive Notch signaling necessary for endothelial repair and long-term vascular integrity. 

## 5. Endothelial Dysregulation Across CVD

Building on the regulatory framework outlined above, convergent evidence suggests that dysregulated endothelial Notch signaling contributes to cardiovascular pathology. The following subsections examine the disease-specific and mechanistic patterns of dysregulated Notch activation, contextualizing the manifestation of persistent activation across distinct CVD contexts.

### 5.1. Atherosclerosis and Coronary Artery Disease

Atherosclerosis exemplifies a well-defined pathological setting of CVD, characterized by persistent endothelial Notch signaling, which contributes directly to both onset and progression. In atherosclerotic lesion-prone arterial regions, exposure to disturbed hemodynamic flow induces endothelial dysfunction, which promotes vascular remodeling, lipid accumulation, and inflammatory cell recruitment [7]. Suppression of laminar shear-aligned Notch signaling within these regions disrupts transcriptional programs that normally function to constrain osteogenic and calcific gene networks through anti-mediators such as the matrix Gla protein (MGP), thus increasing susceptibility to plaque calcification and enhancing bone morphogenic proteins (BMP)-mediated osteogenic gene expression, further predisposing vessels to vascular wall stiffening and pathological remodeling [7,25].

In congruence to the observed flow-mediated effects, in vivo analyses indicate that endothelial Notch signaling is not uniformly suppressed in atherosclerosis but is instead activated at sites with established plaque formation. Immunohistochemical analysis of human and murine aortic tissue reveals a pronounced upregulation of Notch receptors (Notch1, Notch3, and Notch4), ligands (JAG1 and DLL4), and canonical downstream targets (Hes1 and Hey1), selectively localized within luminal endothelial cells overlying established plaque regions, but not in adjacent non-atherosclerotic regions [51]. With persistent activation of endothelial Notch signaling, endothelial proliferative capacity is impaired and induces a senescence-associated phenotype characterized by altered morphology, growth arrest, increased senescence-associated β-galactosidase activity, and reduced telomerase activity [51]. In vivo studies additionally indicate that such a persistent Notch-mediated senescent state is coupled with activation of pro-inflammatory transcriptional programs, characterized by the increased expression of key mediators of leukocyte recruitment and vascular inflammation, including RANTES, ICAM-1, IL-6, IL-8, and IL-1α [49]. Notably, Notch-driven inflammatory signaling within endothelial cells is sufficient to facilitate an early stage of atherogenesis through enhanced monocyte transendothelial migration, via active production of chemokines rather than through junctional destabilization alone. IL-6 has been noted as a partial yet significant regulator of this increased chemokine production response, as IL-6 neutralization attenuates monocyte transmigration induced by Notch signaling across endothelial monolayers [51]. Enforced activation of endothelial Notch signaling thereby facilitates recruitment of leukocytes through both chemotactic and endothelial structural remodeling. Dysregulated Notch signaling in these lesion-prone vascular regions sustains inflammatory responses and endothelial dysfunction, promoting a permissive environment for the recruitment of leukocytes and infiltration of lipids. Attenuated hemodynamic flow further compromises ligand-dependent Notch signaling, shifting from DLL4-mediated stabilization toward preferential JAG1 activation and amplifying endothelial activation [25]. These observed ligand engagement shifts correlate with increased EndMT and extracellular matrix production, contributing to both progression of plaque and fibrotic remodeling. 

In atherosclerotic environments, EndMT promotes plaque growth, fibrotic remodeling, and increased rigidity of vascular architecture through the generation of fibroblastic-like and endothelial matrix-producing cells that potentiate lesion expansion and structural stiffening [7]. Consistent with the inflammatory phenotypes and endothelial senescence observed because of these atherosclerotic EndMT- and Notch-driven endothelial mechanisms, genetic association analyses were conducted on a large cohort of patients with coronary artery disease, revealing significant associations between single-nucleotide polymorphisms and coronary artery disease across multiple genes associated with the Notch pathway [51]. Such integrative associations position endothelial Notch signaling as a mechanistic influence on clinical expression of atherosclerosis. Coronary artery disease (CAD) is a principal clinical manifestation of atherosclerosis, arising from atherosclerotic plaque formation and remodeling within the coronary arterial wall, compromising myocardial perfusion and precipitating ischemic injury [52]. Consequently, CAD is determined by both cumulative burden and composition of plaque, endothelial dysfunction, and inflammatory activated signaling within the coronary arteries. Within coronary artery vasculature, endothelial Notch signaling exhibits a heightened sensitivity to hemodynamic flow disturbance, lipid-mediated metabolic stress, and inflammatory cytokine exposure. 

Dysregulated expression of Notch signaling ligands alongside impaired laminar shear-responsive Notch activation is a distinguishable characteristic in CAD, linking these changes to adverse clinical outcomes that disrupt normal functioning Notch-dependent transcriptional programs. In addition to its effects on endothelial barrier function, dysregulated Notch signaling within CAD interfaces with post-transcriptional and inflammatory regulatory networks, including microRNA-mediated modulation of pathway activity and downstream effectors [52]. Converging data reinforces endothelial Notch signaling as a driver of maladaptive vascular remodeling, contributing to the progression of calcified plaque and coronary artery disease in both experimental murine models and population-level human findings.

### 5.2. Vascular Aging and Endothelial Senescence

Vascular aging, a major independent risk factor of atherosclerosis and CAD, reflects a progressive deterioration of regulatory endothelial plasticity and vascular structure, where the functional capacity to integrate inflammatory, metabolic, and mechanical signals becomes increasingly constrained. Under these conditions, vascular aging disrupts the mechanosensitive calibration of endothelial Notch signaling through alteration of laminar shear responsiveness and stress-associated transcriptional outputs, thereby impairing the ability of Notch to regulate vascular stress-responsive signaling and endothelial cellular lifespan.

Mechanistic evidence indicates that endothelial Notch signaling regulates cellular lifespan through activation of the p16^INK4a^-dependent senescence pathway. Within human endothelial cells, genetic inhibition of Notch signaling drives premature senescence characterized by p16^INK4a^ induction, growth arrest, and increased β-galactosidase activity [53]. Conversely, within the normal physiological context, activation of endothelial Notch functions to promote the expression of the transcriptional regulator inhibitor of DNA binding-1 (Id1) and MAP kinase phosphatase-1 (MKP1), thereby suppressing p38 MAPK-dependent stabilization of p16^INK4a^ and delaying endothelial senescence through a telomere-independent mechanism that sustains replicative lifespan and proliferative capacity [53]. Loss of this protective stress-adaptive axis promotes sustained p38-dependent stabilization of the p16^INK4a^ pathway, accelerating the onset of progressive endothelial aging. 

Notably, aging-associated Notch dysregulation does not reflect comprehensive pathway silencing. Instead, Notch signaling in an aged endothelium remains transcriptionally active but becomes uncoupled from environmental feedback, resulting in functional reprogramming and altered downstream target outputs [53]. Quantitative PCR array and immunoblot analyses of aged human endothelial cells demonstrated elevated expression of the Notch target gene HeyL relative to young cells. This convergence suggests that chronic endothelial Notch activation represents a distinguishable feature different than that exhibited in regulated ligand-dependent Notch signaling, the latter of which shifts transcriptional programming toward inflammatory amplification and architectural degradation. 

MicroRNA profiling data demonstrate that pathologically associated networks of miRNA selectively modulate post-transcriptional control of Notch regulators, mediators, receptors, and ligands, leading to sustained Notch signaling in altered transcriptional networks [52]. At the functional level, the impaired regulatory state of endothelial Notch aligns with hallmark features of vascular aging, including nitric oxide bioavailability, compromised shear-responsive transcriptional programs, and lowered thresholds for inflammatory activation within the endothelium. Continued disruption of necessary regulatory frameworks provides a mechanistic explanation for age-associated CVD vulnerability.

### 5.3. Pulmonary Arterial Hypertension

Pulmonary arterial hypertension (PAH) refers to a progressive vascular disorder characterized by chronic elevation of pulmonary arterial pressure and vascular resistance. In untreated cases, sustained expression of PAH pathology results in progressive intimal, medial, and adventitial thickening and distal vascular obliteration, ultimately leading to right ventricular heart failure and reduced survival expectancy. Pathognomonic features of PAH include sustained vasoconstriction, endothelial dysfunction, and pulmonary vascular remodeling, manifesting as a direct consequence of increased pulmonary vascular endothelial cells; decreased apoptosis of pulmonary arterial smooth muscle and endothelial cells; and increased EndMT, which drives differentiation of endothelial cells into highly proliferative fibrogenic myofibroblast-like cells, directly contributing to neointimal formation and luminal degradation [54]. 

Accordingly, endothelial Notch signaling has emerged as a critical but paradoxical regulator of pulmonary vascular homeostatic responses, influencing physiological, pathogenic, and reparative states dependent on a mechanosensitive cellular context. Within this framework, endothelial Notch signaling serves as a context-dependent regulator of vascular regeneration and coordination of angiogenesis, facilitating the recruitment of smooth muscle cells and proliferation of endothelial cells. When this mechanosensitive regulatory balance is disrupted, pathological amplification of Notch signaling shifts its function towards maladaptive hyperproliferation states that contribute to pulmonary fibrosis, hypertension, and related fibrotic diseases. Specifically, upregulation of Notch1 in endothelial cells and Notch3 in pulmonary arterial smooth muscle cells is a characteristic feature observed in both experimental models of hypoxia-induced pulmonary hypertension and in human PAH [54]. 

Concurrently, pulmonary vascular remodeling within a PAH context reflects an impaired resolution of endothelial regenerative signaling in response to vascular injury formation [54]. Under homeostatic conditions, direct contact between smooth muscle and endothelial cells initiates a BMPR2-dependent endothelial program regulated through Notch1 activation. BMPR2 signaling functions to induce JNK signaling, promote the deposition of extracellular matrix collagen IV, and activate integrin-linked kinase (ILK) to stabilize the γ-secretase complex and sustain downstream transcriptional activation and processing of Notch1. To support vascular integrity following injury, Notch1 functions to promote endothelial cell regeneration and survival through activation of glycolytic regulators required for endothelial monolayer restoration consistent with its homeostatic roles described earlier in this review [54]. As Notch-mediated endothelial repair persists, re-establishment of a continuous endothelial monolayer following injury restores contact between smooth muscle and endothelial cells, which activates Notch3 signaling in pulmonary arterial smooth muscle cells. Subsequently, signaling from the Notch3 intracellular domain (N3ICD) promotes transcriptional programs that facilitate medial smooth muscle hypertrophy as well as directly modulate plasma membrane calcium channel activity to increase intracellular calcium influx and sustain pulmonary vasoconstriction [54]. 

Genetic studies further characterize cell-specific divergence and non-interchangeable roles between Notch homologs in pulmonary vascular remodeling, where Notch3-deficient mice are protected from hypoxia-induced pulmonary hypertension, whereas compared to when deficient in endothelial Notch1, disease symptoms persist and severity is exacerbated. Notably, altered signaling of BMPR2 disrupts the regulatory balance of Notch activation, with further amplification of discussed effects from crosstalk between HIF, Notch, and metabolic regulators including PFKFB3 [54]. In PAH, endothelial Notch signaling shifts from a tightly regulated transient mechanism to a sustained driver of pathological vascular remodeling through cell-specific divergence between endothelial Notch 1 and smooth muscle Notch 3 signaling as well as disrupted coordination of signal integration across the vessel wall. This function divergence highlights why global inhibition of the Notch pathway is likely to be unsuccessful in yielding a therapeutic benefit, emphasizing the critical need for condition-specific precision-based strategies to restore essential Notch regulatory capacity and maintain its homeostatic roles.

### 5.4. Endothelial Notch Dysregulation in Arteriovenous Malformations (AVMs)

Arteriovenous malformations (AVMs) are characterized by aberrant direct arterial–venous connections that bypass the capillary bed, producing high-flow, low-resistance shunts and profound hemodynamic imbalance. Although AVMs are often considered developmental vascular anomalies, accumulating evidence positions endothelial Notch signaling as a central regulator of arteriovenous identity whose dysregulation underlies AVM initiation and progression. Canonical Notch1–DLL4–HEY signaling is essential for arterial specification and maintenance of arteriovenous segregation. Endothelial-specific loss of Notch1, DLL4, or RBPJ in murine models results in spontaneous AVM formation, characterized by loss of capillary intermediates, venous arterialization, and vessel dilation [16,55,56]. 

Conversely, constitutive endothelial NICD activation is likewise sufficient to induce AVM-like phenotypes, demonstrating that both insufficient and excessive Notch signaling disrupt vascular hierarchy, underscoring the requirement for tightly calibrated, dynamically regulated Notch activity [57,58]. AVM pathogenesis is further reinforced by crosstalk between Notch and the BMP/TGF-β signaling axis, particularly in the setting of hereditary hemorrhagic telangiectasia (HHT) [59,60]. Loss-of-function mutations in ALK1 (ACVRL1) or ENG (endoglin), hallmark genetic drivers of HHT, impair endothelial responsiveness to shear stress and disrupt BMP9/10 signaling, leading to secondary dysregulation of Notch activity [61,62,63,64]. In ALK1- or ENG-deficient endothelium, Notch signaling becomes uncoupled from normal flow-dependent restraint, permitting inappropriate arterial gene expression, endothelial hyperproliferation, and failure of capillary specification [61]. Genetic epistasis studies demonstrate that Notch acts downstream and in parallel with ALK1 signaling to integrate hemodynamic cues and enforce arteriovenous fate decisions; disruption of this coordination precipitates AVM formation [59,60]. 

Hemodynamic forces further amplify Notch dysfunction in established AVMs. Once arteriovenous shunts form, venous endothelial cells are exposed to arterial-level shear stress, driving mislocalized and persistent Notch activation that lacks proper ligand bias or signal termination. Unlike physiological laminar shear-induced Notch activation, which promotes endothelial quiescence, AVM-associated shear reinforces pathological arterialization of venous endothelium and lesion expansion [62,63,64]. Aberrant DLL4/JAG1 balance further compromises lateral inhibition and endothelial patterning, suppressing capillary differentiation and stabilizing the AVM phenotype [16].

These findings underscore that AVMs exemplify a disease state where endothelial Notch signaling fails as a mechanosensitive fate regulator, not through uniform hyperactivation or suppression, but through loss of spatial, temporal, and hemodynamic contextual control. These insights reinforce the broader theme of this review: therapeutic strategies for AVMs must aim to restore calibrated, flow-responsive endothelial Notch signaling, rather than indiscriminate pathway inhibition, to preserve vascular stability while limiting pathological remodeling.

### 5.5. EC Notch Dysregulation Across Heart Failure Phenotypes: Heart Failure with Preserved Ejection Fraction (HFpEF) and Heart Failure with Reduced Ejection Fraction (HFrEF)

While pulmonary hypertension exhibits dysregulated endothelial Notch signaling within the pulmonary circulation, heart failure represents a distinct but mechanistically convergent disease class where maladaptive endothelial signaling causes systemic dysfunction rather than solely in the localized vascular obstruction, as exhibited in PAH. The most common subclasses of heart failure include heart failure with preserved ejection fraction (HFpEF) and heart failure with reduced ejection fraction (HFrEF), and while both entities differ in upstream stressors and downstream cardiac responses, endothelial Notch dysfunction has become relevant as a shared feature amongst both forms of heart failure and contribute to vascular inflammation, stiffness, and vascular integrity [65,66]. 

Heart failure with preserved ejection fraction (HFpEF) is distinguished by the stiffening of heart muscles and thus directly to left ventricular blood filling abnormalities despite pumping ability (ejection fraction) and systolic function appearing to be normal [66]. In this expression, endothelial dysfunction, characterized by reduced nitric oxide bioavailability, increased OS, and enhanced endothelial stiffness, arises early and systemically through chronic cardiometabolic comorbidities, including obesity, hypertension, diabetes, and aging [66]. Comparatively, endothelial dysfunction associated with heart failure with reduced ejection fraction (HFrEF) is defined by reduced left contractile function and impaired systolic output because of loss of cardiomyocytes, neurohumoral activation, and ischemic injury [66]. Following myocardial injury, Notch signaling may initially support endothelial survival and angiogenic repair for a short period of time; however, prolonged relevant stressors promote impaired vasodilatory capacity, vascular dysfunction, and inflammatory amplification that exacerbate systolic failure and vascular remodeling [66]. While the role of Notch signaling in heart failure phenotypes remains incompletely understood, both HFpEF and HFrEF serve as illustrations of how endothelial Notch signaling functions as a regulatory axis and further emphasizes the need to explore therapeutic approaches that target mechanosensitive responsiveness as compared to global Notch suppression and indiscriminate pathway inhibition.

## 6. Therapeutic Aims of Dysregulated Endothelial Notch Activation in CVD

**Figure 4:**
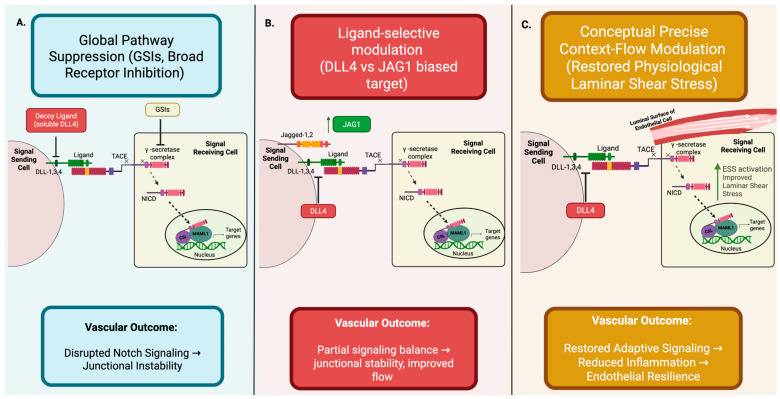
Comparative visualization of therapeutic approaches targeting endothelial Notch signaling in CVD. Global Notch pathway suppression (**A**), through early interventions using GSIs and broad receptor inhibition, disrupts physiological endothelial Notch signaling, subsequently leading to downstream effects of impaired junctional stability and systemic vascular toxicity. In the center image (**B**), ligand-selective modulative therapies, such as through DLL4- or JAG1-preferential biased targeting, allow partial perseveration of adaptive Notch signaling to be retained and further improved through localized endothelial stabilization; however, these strategies are limited due to incomplete specificity. While still conceptual and in development, precise context-dependent modulation of endothelial Notch signaling in CVD (**C**) represents a promising avenue to restore physiological laminar shear stress responsiveness and re-engage mechanosensitive Notch signaling without compromising non-inflamed vascular regions. This schematic is partially adapted from [10], with appropriate modifications to reflect endothelial Notch-specific signaling and therapeutic design. The lowercase ‘x’ denotes the site of TACE-mediated proteolytic cleavage in the Notch signaling pathway. Figure created with Biorender.com.

Advances in the understanding of endothelial Notch signaling as a finely tuned mechanosensitive regulatory axis of vascular homeostasis have not only broadened comprehensive understanding of CVD but also highlighted significant therapeutic potential in targeting this pathway under the persistent pathological context. In healthy vasculature, Notch activity is regulated through metabolic inputs, hemodynamic forces, regenerative and adaptive capacity, and inflammatory cues to maintain endothelial integrity. In CVD, the regulatory framework of Notch signaling is distorted by altered hemodynamic flow, inflammatory cytokines, and metabolic stressors, which reinforce mislocalized activation that becomes functionally disengaged from coordinated endothelial stabilization pathways and physiological feedback (Figure 4). 

The context-dependent nature of regulatory Notch signaling poses a significant and complex challenge for therapeutic intervention, as uniform inhibitory approaches neglect laminar flow-aligned signaling and localized pathological activation within inflamed or shear-disturbed vascular regions. Consequently, global inactivation may progressively disrupt physiological vascular stability while ineffectively addressing pathological remodeling. Effective therapeutic approaches of dysregulated endothelial Notch necessitate targeting both environmental responsiveness and the capacity of endothelial Notch signaling to appropriately scale its intensity and duration of resolution to upstream mechanical, inflammatory, and metabolic cues.

### 6.1. Broad Notch Inhibition Strategies

Initial therapeutic efforts targeting pathological states of the endothelial Notch pathway were widely centered around global pathway suppression, notably using γ-secretase inhibitors (GSIs) [67]. GSIs inhibit S3 cleavage of the Notch receptor, preventing NICD release and nuclear translocation, thereby resulting in broad suppression of downstream transcriptional signaling pathways [67]. While expressional Notch activity was suppressed, GSIs posed substantial limitations in CVD contexts due to cellular unspecificity and dose-limiting toxicities [68]. Additional therapeutic attempts have deployed antibody-based strategies through specific receptor modulation, specifically with antibodies that hold the functional capacity to induce ligand-independent activation of Notch receptors, such as Notch3. While these studies reinforced that selectively targeting individual Notch receptors is possible, the same lack of spatial and temporal control as well as systemic delivery continues to constrain clinical applicability [68]. 

With similar unintentional therapeutic outcomes, targeting the DLL4-Notch1 axis emerged as another early effort pursued to constrain pathological angiogenesis through more specific monoclonal antibodies (mAbs) [69,70]. DLL4-selective antibodies effectively disrupt differentiation of endothelial cells as well as downstream Notch signaling, consequently leading to excessive but ineffective angiogenic sprouting and impaired vascular patterning [69]. While these therapeutic efforts exhibited significant tumor-suppressive effects within preclinical models, the translational application of these findings is limited by adverse effects associated with the global disruption of Notch signaling, including systemic vascular dysfunction [69]. 

Effective communication between DLL4 and Notch1 is critical in physiological contexts, as it is necessary to maintain lumen stability and appropriate categorization of vasculature. Preclinical studies further demonstrated that blocking DLL4 disrupts ligand’s endothelial tip-stalk cell patterning through a decrease in VEGFR2/3 expression consequent of Notch repression [69]. While the efficacy of this approach has been proven in minimizing the vascularization of tumor growth through the induction of excessive non-productive angiogenesis, translational applicability in mediating CVD is significantly limited. DLL4-mediated Notch signaling is imperative to maintaining junctional stability, structuring endothelial patterns, and overall efficiency of perfusion within the adult vasculature, and thus, the global disruption of DLL4-mediated Notch activation poses significant risks to the physiological vascular integrity of non-diseased endothelial tissue. The downstream effects of the disruption of the crosstalk between DLL4 and Notch1 resulted in excessive, irregular, and disorganized endothelial sprouting, leading to poorly perfused vascular networks and compromised vascular structure [69].

### 6.2. Ligand-Selective and Endothelial-Targeted Approaches

Building on the use of the DLL4 ligand as a potential therapeutic target in CVD, more recent advances have shifted from global pathway suppression to ligand-specific modulation in endothelial Notch signaling. Specifically, these approaches capitalize on the observed functional divergence of DLL4- and JAG1-mediated Notch signaling outputs. While DLL4-Notch1 signaling restricts endothelial tip cell specification, JAG1-mediated signaling promotes endothelial survival, junctional stability, and adaptive vascular reconstruction in physiological contexts [16]. 

Concurrently, development of endothelial-targeted delivery strategies has been engineered to spatially restrict Notch pathway modulation solely to affected diseased vascular beds. Utilizing hyaluronan-based nanoparticles within ApoE^−/−^ mice, early atherosclerotic regions were characterized by disrupted VE-cadherin-based adherens junctions with enlarged discontinuous intercellular gaps, enabling effective transport of the engineered nanoparticle across the plaque covered endothelium. Comparatively, in the observed effects of junctional stabilization during plaque progression, a significant decrease in endothelial permeability and nanoparticle accumulation is observed due to increased continuity of VE-cadherin networks [71].

### 6.3. Spatial and Hemodynamic Precision: Targeting Disturbed Flow Regions

Endothelial Notch signaling is a pathway dictated by local hemodynamic flow and operates as an environmentally regulated conservative integrator of mechanical, molecular, and inflammatory cues; therefore, spatially targeted therapies hold a particularly promising approach in relieving the complex clinical manifestation of dysregulated Notch signaling. In vascular regions associated with oscillatory laminar shear stress, particularly exhibited in arterial bifurcations and curvatures, dysregulated Notch signaling is not only apparent but also accompanied by impaired junctional organization, inflammatory priming, and increased susceptibility to atherosclerotic phenotypes. Within these stress-enhanced domains, selective regeneration of mechanosensitive integrators of the Notch pathway occurs to redirect transcriptional programs while preserving effective protective signaling in vascular segments exposed to laminar shear stress. 

Recent advances in vascular engineering and mechanotherapy directly reinforce this paradigm by supporting the recalibration of endothelial signaling states through controlled modulation of local endothelial shear stress (ESS). The multidimensional optimization of stent design includes adjustments made to strut thickness, geometric strut profiles, and inter-struct spacing to modulate endothelial shear stress, where emerging evidence demonstrates that thinner struts, optimized spacing, and streamlined profiles optimize spacing and improve hemodynamics to reduce low and shear stress regions occurring post stent-implantation and thus promote the restoration of a more uniform shear stress environment along the stented vessel segment [72]. This hemodynamic flow restoration is associated with improved angiogenesis of endothelial cells, reduced inflammatory activation, and neointimal hyperplasia suppression, demonstrating that flow correction to physiological shear stress serves as a direct regulator of endothelial function. Restoration of physiological ESS states is associated with the re-engagement of shear-dependent Notch1 signaling at endothelial junctions and thus promotes transcriptional programs that modulate inflammatory activation and endothelial cardiovascular remodeling within these disturbed regions.

## 7. Current Gaps in Understanding of Endothelial Notch Mechanism and Future Directions

Emerging advances in delineating endothelial Notch activation as a mechanosensitive regulator of vascular homeostasis have shifted the paradigm from global pathway inhibition to precise modulation of Notch signaling, specifically, exploring influences such of spatial delivery, ligand specificity, and hemodynamic flow modulation as potential avenues for therapeutic efforts. However, despite these developments, several critical knowledge gaps continue to limit translational application in chronic cases of CVD. While progressive dysfunction of physiological Notch signaling is understood to lead to maladaptive remodeling as well as substantial compromise in the integration of hemodynamic forces with metabolic and inflammatory cues within a vascular context, the temporal and quantitative parameters influencing this integration remain largely undefined in vivo. In particular, the field lacks a unified framework for how the influence of shear stress profiles, cytokine exposure, and the inflammatory and metabolic microenvironment shape endothelial Notch transcriptional outputs across vascular beds, and how these inputs are hierarchically integrated under physiological and pathological conditions. 

A major unresolved challenge lies in the phenotypic heterogeneity of endothelial Notch responsiveness, where identical inflammatory or mechanical inputs prompt divergent signaling outputs depending on vascular beds, endothelial age, ligand distribution, and transcriptional capacity. Age-associated disease models exhibit gradual alterations in the flexibility of Notch signaling as a mechanostat, thereby reducing its capacity to dynamically scale its signal, as redirected ligand utilization and delayed termination of signals constrain endothelial Notch responsiveness [73,74]. Additional findings indicate sex-specific difference within endothelial inflammatory and mechanotransductive signaling; however, the underlying biochemical variables, including the full comprehensive understanding of Notch pathway involvement, remain incompletely defined [74]. Of equal importance in current limitations within the field is the lack of quantitative thresholds distinguishing between adaptive and maladaptive Notch activation signaling. Current frameworks position Notch activity within qualitative terms, without determining if pathological outcomes reflect aberrant signal intensity, ligand-specific preference to DLL4 or JAG1, activation kinetics, and compromised signal termination upon exposure to vascular stress [4,72]. From a therapeutic standpoint, the development of treatment strategies remains in the early stages, particularly in engineering precision-based endothelial Notch modulation.

Despite ongoing developing efforts centered around ligand-specific modulation, restrictive endothelial-based delivery systems, and hemodynamic flow-restorative interventions, the long-term consequences on immune regulation, vascular remodeling, and tissue reparative capacity remain insufficiently characterized. Taken together, failure to account for the contextual diversity of endothelial Notch signaling risks a far too reductionist approach in targeting disease mechanisms and undermines the need for precision-based modulation. Establishing quantitative benchmarks moving forward should be at the forefront of future direction, specifically those that distinguish flow states, vascular beds, and disease stages across physiological and chronically maladaptive activation states. Addressing current knowledge gaps will require integrative experimental designs that combine biomechanical modeling, single-cell-resolved transcriptional and epigenetic profiling, and longitudinal assessment of functional assessments. Determining whether CVD outcomes arise from prolonged temporal persistence, dysregulated signal resolution or intensity, and ligand bias will be critical for translating mechanistic understanding into both safe and clinically actionable therapeutic intervention models.

## 8. Conclusions

This review synthesizes current evidence framing endothelial Notch signaling as a critical regulator of cardiovascular homeostasis, functioning as a mechanosensitive signaling axis that integrates hemodynamic forces with metabolic and inflammatory signaling. Across physiological contexts, highly conserved and ligand-dependent Notch signaling supports endothelial quiescence, adaptive remodeling, regenerative capacity, and barrier integrity; however, in exacerbated CVD states, including atherosclerosis, heart failure, pulmonary hypertension, and coronary artery disease, this regulatory competence becomes compromised. Progressive disruption of Notch signaling manifests as spatially mislocalized, prolonged, or preferential ligand-biased activation that disengages from vascular endothelial-stabilizing programs and homeostatic conditions. 

Further, disturbed flow, metabolic stress, endothelial aging, and chronic inflammation contribute to the degradation of this adaptive and regenerative capacity and further promote EndMT, pro-inflammatory transcriptional outputs, and maladaptive vascular remodeling. Notably, these pathological outcomes arise not solely from pathway hyperactivation but also from impaired or misdirected resolution of activated signaling, challenging prior simplistic understanding of Notch-driven disease.

Ultimately, endothelial Notch signaling should not be conceptualized to be therapeutically addressable through simple on/off activation or inhibition but reframed as a regulatory signaling integration hub whose output is influenced by metabolic, inflammatory, and hemodynamic flow context. Accordingly, therapeutic efforts must be framed to address maladaptive cardiovascular expression associated with failure of contextual control of Notch signaling. Disease phenotypes more accurately reflect shifts in activated signaling dynamics rather than complete pathway overactivation, highlighting the fundamental limitations of global Notch inhibition as the centered therapeutic approach. The clinical relevance of endothelial Notch signaling derives from its fundamental mechanosensitive endothelial plasticity, positioning it to be best suited in context-dependent modulation. Effective translation of mechanistic understanding will require defined quantitative parameters that distinguish between physiological and pathological activation of Notch signaling across various CVD stages, vascular beds, ages, and patient populations.

With this focus, precision-based efforts work to correct disease-associated expression through ligand-biased targeting, endothelial restricted delivery, and regeneration of hemodynamic flow conditions, all while preserving physiological signaling. Advancing progress in endothelial Notch-directed cardiovascular therapy requires integrative approaches that account for the complex interplay of endothelial signaling context, phenotypic variability, signaling thresholds, and spatial regulation. Future therapeutic success will depend on redefining Notch not as a target to globally suppress but as a dynamic system whose maladaptive signaling requires precise, context-dependent recalibration, thereby positioning it as both an effective cardiovascular intervention as well as a means to restore vascular integrity and functional resistance.

## Data Availability

No new data were created or analyzed in this review article.

## Abbreviations

The following abbreviations are used in this manuscript:

ADAM10	A Disintegrin and Metalloprotease 10
ALK1	Activin Receptor-Like Kinase 1
APH1	Anterior Pharynx Defective 1
AVM	Arteriovenous Malformation
BMP	Bone Morphogenetic Protein
CSE	Cystathionine γ-lyase
CVD	Cardiovascular Disease
DLL	Delta-Like Ligand
EC	Endothelial Cell
EndMT	Endothelial-to-Mesenchymal Transition
ENG	Endoglin
eNOS	Endothelial Nitric Oxide Synthase
ESS	Endothelial Shear Stress
GSIs	γ-Secretase Inhibitors
H_2_S	Hydrogen Sulfide
HFpEF	Heart Failure with Preserved Ejection Fraction
HFrEF	Heart Failure with Reduced Ejection Fraction
HES	Hairy and Enhancer of Split
HEY	Hairy/Enhancer-of-Split Related with YRPW Motif
HHT	Hereditary Hemorrhagic Telangiectasia
iNOS	Inducible Nitric Oxide Synthase
JAG	Jagged Ligand
KLF2	Krüppel-Like Factor 2
KLF4	Krüppel-Like Factor 4
mAbS	Monoclonal Antibodies
NF-κB	Nuclear Factor Kappa B
NICD	Notch Intracellular Domain
NO	Nitric Oxide
PAH	Pulmonary Arterial Hypertension
PEN2	Presenilin Enhancer 2
PI3K	Phosphoinositide 3-Kinase
RBPJ	Recombination Signal Binding Protein for Immunoglobulin Kappa J Region
ROS	Reactive Oxygen Species
TAZ	Transcriptional Co-activator with PDZ-binding Motif
TGF-β	Transforming Growth Factor Beta
VE-cadherin	Vascular Endothelial Cadherin
VEGF	Vascular Endothelial Growth Factor
Wnt	Wingless/Integrated signaling pathway
YAP	Yes-Associated Protein
